# A feasibility study for in vivo treatment verification of IMRT using Monte Carlo dose calculation and deep learning-based modelling of EPID detector response

**DOI:** 10.1186/s13014-022-01999-3

**Published:** 2022-02-10

**Authors:** Jun Zhang, Zhibiao Cheng, Ziting Fan, Qilin Zhang, Xile Zhang, Ruijie Yang, Junhai Wen

**Affiliations:** 1 Department of Biomedical Engineering, School of Life Science, Beijing Institute of Technology, Beijing, China; 2 Department of Radiation Oncology, Peking University Third Hospital, Beijing, China

**Keywords:** Monte Carlo, PRIMO, Deep learning, EPID, In vivo verification

## Abstract

**Background:**

This paper describes the development of a predicted electronic portal imaging device (EPID) transmission image (TI) using Monte Carlo (MC) and deep learning (DL). The measured and predicted TI were compared for two-dimensional in vivo radiotherapy treatment verification.

**Methods:**

The plan CT was pre-processed and combined with solid water and then imported into PRIMO. The MC method was used to calculate the dose distribution of the combined CT. The U-net neural network-based deep learning model was trained to predict EPID TI based on the dose distribution of solid water calculated by PRIMO. The predicted TI was compared with the measured TI for two-dimensional in vivo treatment verification.

**Results:**

The EPID TI of 1500 IMRT fields were acquired, among which 1200, 150, and 150 fields were used as the training set, the validation set, and the test set, respectively. A comparison of the predicted and measured TI was carried out using global gamma analyses of 3%/3 mm and 2%/2 mm (5% threshold) to validate the model's accuracy. The gamma pass rates were greater than 96.7% and 92.3%, and the mean gamma values were 0.21 and 0.32, respectively.

**Conclusions:**

Our method facilitates the modelling process more easily and increases the calculation accuracy when using the MC algorithm to simulate the EPID response, and has potential to be used for in vivo treatment verification in the clinic.

## Introduction

Intensity-modulated radiation therapy (IMRT) and volumetric-modulated arc therapy (VMAT) technologies can control the irradiation area more accurately, ensure that the tumour target receives higher and more conformal doses, reduce the effects on surrounding normal tissues or prevent unnecessary radiation, and these technologies are becoming increasingly common in radiotherapy [1]. Compared with traditional three-dimensional conformal therapy, IMRT is more complicated, with a greater probability of errors in radiotherapy. Due to physical conditions such as the shape and location of human organs, as well as the working stability and repeatability errors of the staff and treatment equipment, differences between the actual irradiation dose and the planned dose may occur. If the dose received in the target deviates significantly from the dose planned by the treatment planning system (TPS), radiation therapy (RT) accidents may occur. As treatment plans become more complex, dose verification is becoming increasingly important in radiotherapy.

Electronic portal imaging device (EPID) has been applied in dose verification by many researchers due to its fast image acquisition speed, high resolution, good linear dose response, long-term stability, and ability to be mounted on the linac [2–4]. Dose verification with EPID is mainly divided into pre-treatment verification [5–7] and in vivo verification [3, 8–11]. Pre-treatment verification is performed before treatment and without the patient present, and in vivo verification is performed during treatment and with the patient present. Pre-treatment verification cannot detect setup errors that occur during treatment, and in vivo treatment verification is more sensitive to possible dose deviations due to changes in tumor size, patient weight and organ motion; thus, in vivo treatment verification is more meaningful. A comprehensive literature review of in vivo verification was presented by Mijnheer et al. [12].

The use of EPID for two-dimensional in vivo treatment verification requires modelling the EPID response to predict the EPID transmission image (TI) during treatment and comparing the predicted and measured TI to verify whether an error occurred during treatment. The traditional method to model the EPID response is mainly based on a physical model [3, 13] and the Monte Carlo (MC) method [6, 10]. The physical model uses a series of measurement data to calculate the scatter kernel of the EPID plane, and the scatter kernel at the central axis is usually used to calculate the scatter value at the off-axis point. Additionally, some empirical correction factors are used. The MC method has always been regarded as the "gold standard" in dose calculation and can accurately simulate the transport properties of various particles. There are two methods to simulate the EPID response using the MC method. One is the full MC technique [14], which simulates photon transport through the linac head and dose deposition in the EPID. The other is to simulate the dose kernel at the EPID plane and then convolve it with the fluence map to obtain the dose deposition in the EPID [10, 15]. Both methods require detailed linac and EPID structures, which are usually industrial secrets and difficult to obtain, and the calculation accuracy is directly related to the modelling accuracy. In addition, the MC calculation process takes a long time and is difficult to use in clinical practice widely. PRIMO is a free MC software that simulates patient dose distribution according to the RT plan and plan computed tomography (CT). It has a friendly graphical user interface and calculation engine based on PENELOPE and the fast dose planning method (DPM) algorithm [16]. More importantly, it integrates several commonly used linac models. The user can select the corresponding linac model without modelling the linac structure and then quickly and easily calculate the dose distribution of the phantom or patient without strong professional knowledge. PRIMO has been applied to the quality assurance of radiotherapy [17–20], but is currently unable to simulate EPID directly.

With the advancement of hardware, deep learning (DL) has made great progress in recent years. Due to its modelling potential, DL based on convolutional neural networks has also begun to be applied in the field of radiotherapy. Different DL networks have been used to predict the dose distribution of patients [21–25]. Zhang et al. [26] used convolutional neural networks to automatically segment clinical targets and organs at risk. Valdes et al. [27] and Zhen et al. [28] used convolutional neural networks to establish an RT toxicity prediction model. Li et al. [29, 30] used a classification regression model to predict patient-specific quality assurance results of VMAT plans. However, there are few studies on in vivo treatment verification using DL.

In this study, to reduce the difficulty of applying MC simulation in clinical treatment verification, we developed a simple and accurate method for two-dimensional in vivo treatment verification based on MC and DL. We used homogeneous solid water instead of the complex EPID. The model trained by the convolutional neural network was used to convert the dose value of solid water calculated by PRIMO into the actual EPID response, and there is no need to code the geometry of the EPID. The advantages of our model are as follows: (1) our method simplifies the process of using the MC algorithm to simulate the EPID response; (2) our method uses a deep learning network to accurately convert the water dose into EPID response; (3) our method can save linac time when performing in vivo dose verification.

## Materials and methods

### Materials

The linac used in the study was a Varian Trilogy Linac (Varian Medical Systems, Palo Alto, CA) equipped with a Millennium 120 multileaf collimator (MLC). The EPID detector (Varian aS1000 flat panel detector) was located 50 cm below the isocentre and covered a field size of 40 cm × 30 cm with a resolution of 1024 × 768 pixels. Dark and flood fields were acquired before the experiment. All measurements were performed using 6 MV X-rays, and the acquisition software was Image Acquisition System 3 (IAS3). The MC environment used is PRIMO (version 0.3.64.1800).

### Methods

In our study, the plan CT was expanded first, and solid water was inserted into the expanded CT as the equivalent EPID at the EPID position to form a combined CT. Then, each field in the RT plan was separated into a new plan. The combined CT and the new plan were imported into PRIMO to simulate the dose distribution of the combined CT, and the dose of the equivalent EPID plane was derived as the input of the DL training model. The measured TI was used as the ground truth to train the DL network. The trained model was used to predict the TI.

**Fig. 1:**
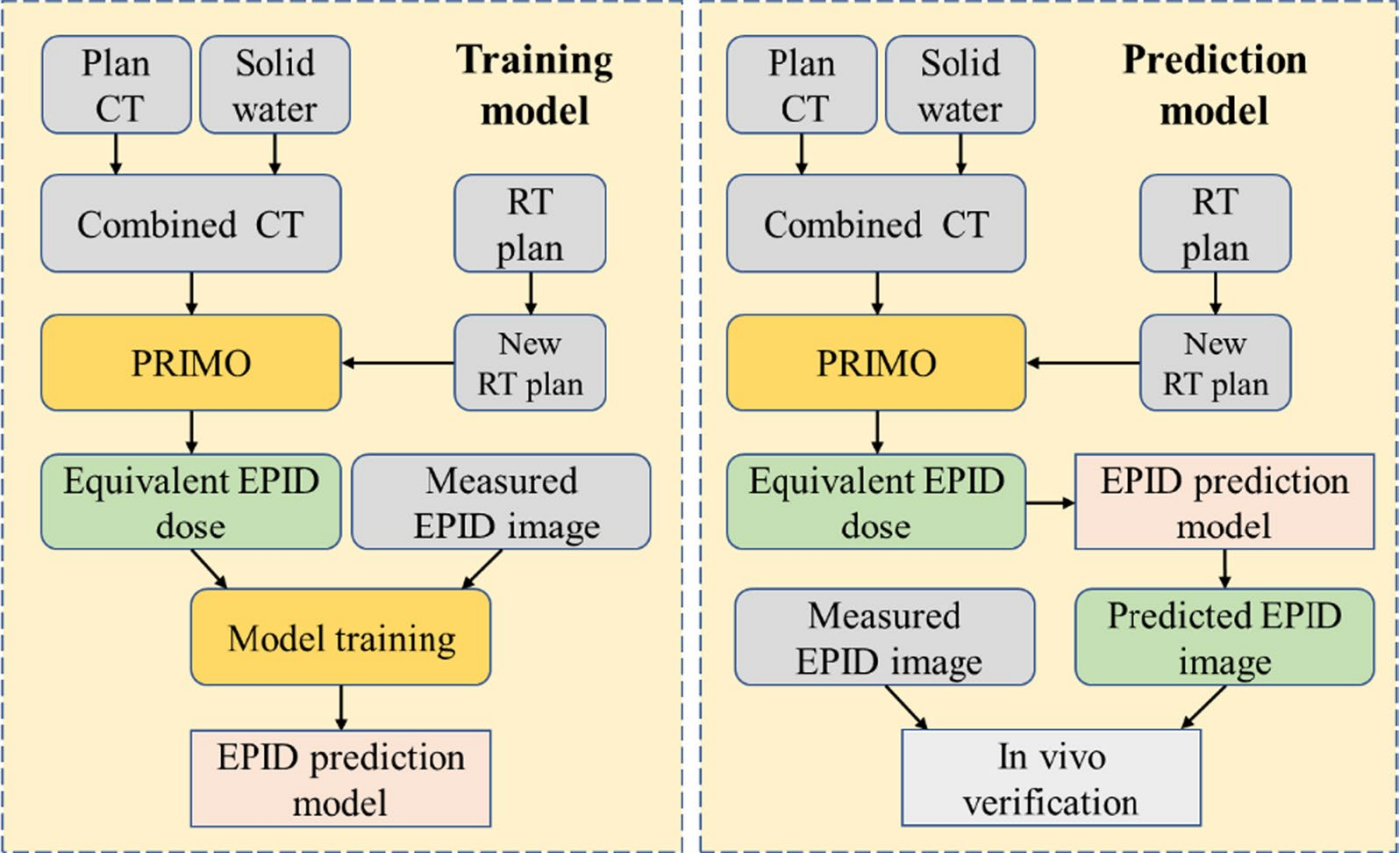
The flow chart of this study. The left box trains the EPID prediction model, and the right box uses the trained model to predict the EPID TI

When treatment verification was performed on phantoms or patients, the combined CT and the new RT plan for each field were imported into PRIMO. PRIMO calculated the dose distribution of the equivalent EPID, and then the TI was predicted by the trained DL prediction model. The predicted TI was compared with the measured TI during treatment for two-dimensional in vivo treatment verification. Figure 1 shows the flow chart of our study.

#### Calculating the equivalent EPID dose distribution

The simulation process in PRIMO is divided into three parts: S1, S2, and S3. S1 simulates the output of the linac head. The corresponding linac model can be selected in the PRIMO library to generate a phase-space file. Alternatively, phase-space files can be imported externally, omitting the simulation of S1. This study selected Varian Clinic 2300 as the linac model and used the external Varian phase-space to calculate the dose. When importing the external phase-space files, PRIMO assumes that they have been tallied at the downstream end of S1.

The S2 part is used to simulate the field configuration. Our study processed the IMRT plan file exported from the TPS using in-house software and selected the Millennium 120 MLC in PRIMO. The EPID rotates with the gantry during treatment, and the position relative to the linac head remains unchanged. Making the equivalent EPID rotate with gantry is difficult to achieve in PRIMO, so we set the angle of each field to 0 and rotated the plan CT in the opposite direction to replace the rotation of the gantry according to the following process:

1. The configuration of each subfield in the RT plan was extracted and written into a new DICOM file; for example, a plan containing three subfields was divided into three new RT plans, and each new RT plan included only one field;
2. The gantry angle of each new plan was set to zero and imported into PRIMO.

**Fig. 2:**
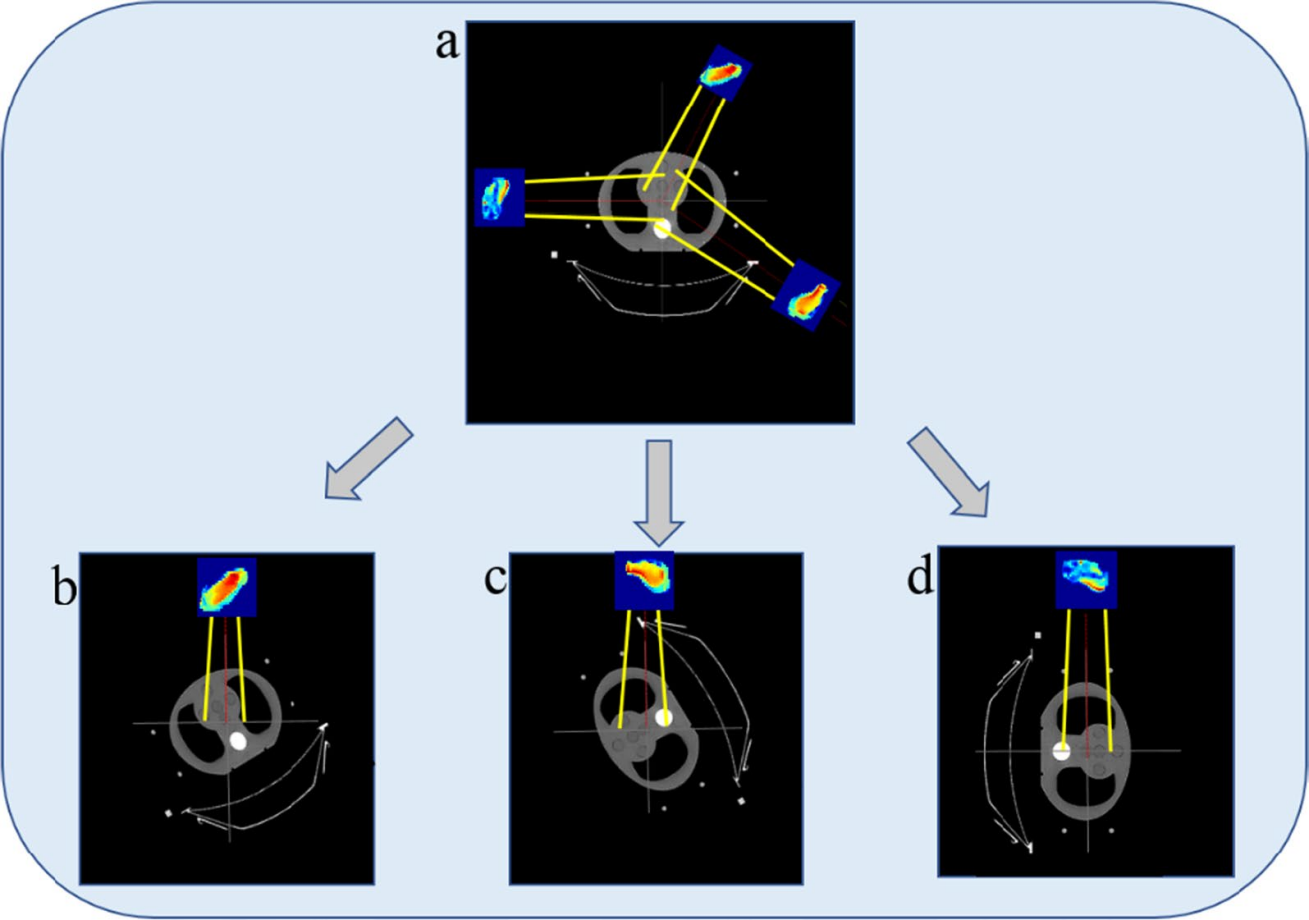
The pre-processing of the RT plan. **a** An RT plan exported from the TPS; **b**, **c** and **d** are the new RT plans after processing

Figure 2a shows an RT plan exported from the TPS containing three fields; the gantry angles are 30°, 120°, and 270°. Figure 2b, c, d are schematic diagrams of the new RT plans imported into PRIMO. Each plan contains one field.

The S3 part is used to simulate the dose distribution of the phantom or patient. We pre-processed the plan CT using the in-house software. The process consisted of the following steps:

1. Replace the CT value of the couch in the plan CT with the recommended value in the TPS;
2. Read the gantry angle of each field from the RT plan, and then rotate the plan CT anti-clockwise by this angle;
3. Extend the plan CT in the axial direction;
4. Place solid water with a thickness of 5 cm (CT = 0 HU) at 47 cm below the isocentre and assign it to air (CT = − 1000 HU) between the couch and solid water;
5. Write the plan CT and solid water as a combined CT into a DICOM file and then import it into PRIMO.

**Fig. 3:**
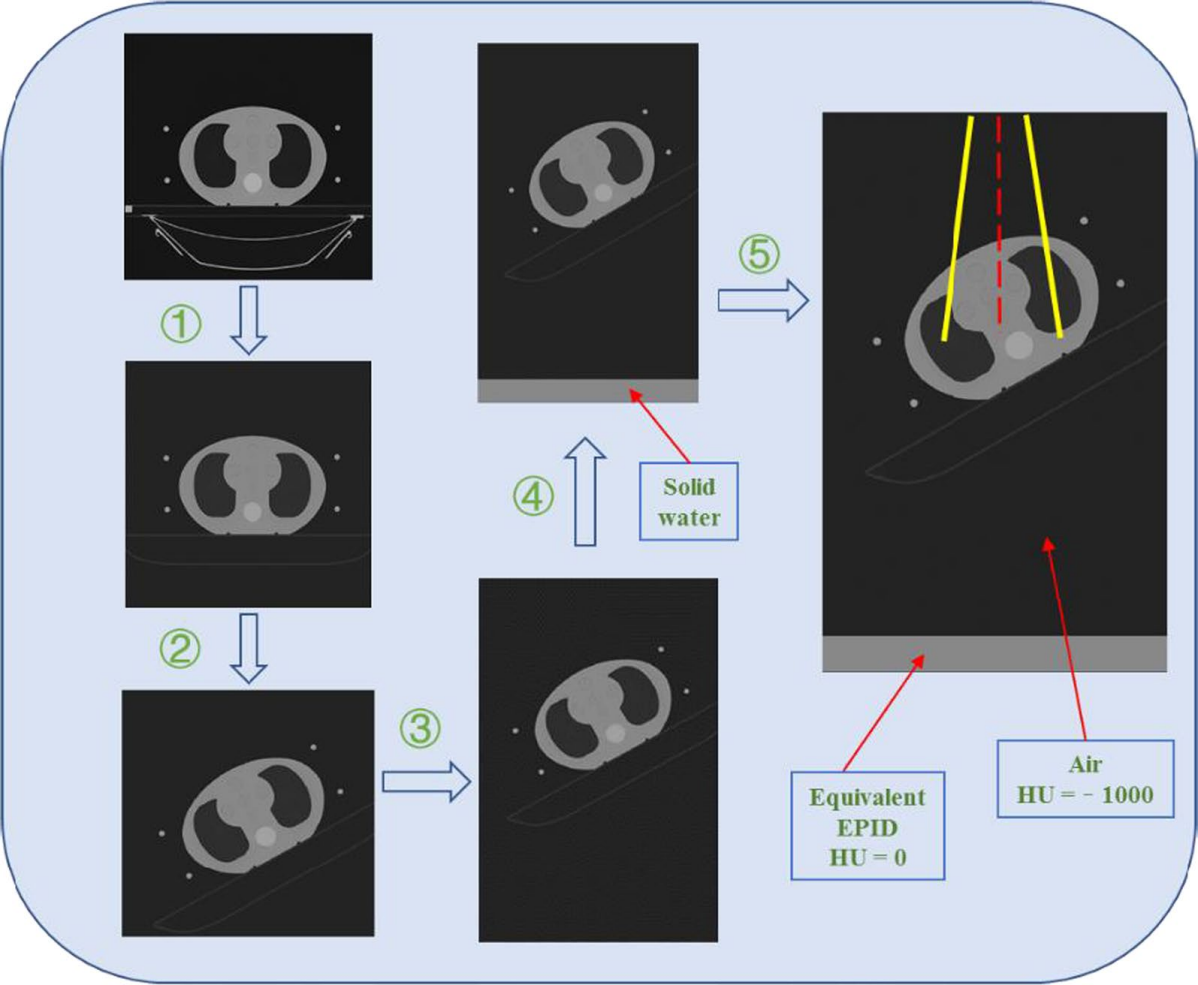
The pre-processing process of the plan CT when the gantry angle is 30°

Figure 3 shows the plan CT's pre-processing process when the gantry angle of the field is 30°. Solid water 50 cm below the isocentre was used as the equivalent EPID, and solid water 3 cm above the equivalent EPID was used as the build-up plate. We used the DPM algorithm provided by PRIMO to calculate the dose distribution. To reduce the statistical uncertainty, the splitting factor was set to 100 as recommended in the PRIMO manual. Each field of the new RT plan was simulated separately. After the simulation, the dose distribution at the coronal plane 50 cm below the isocentre was derived from PRIMO.

#### Deep learning network

**Fig. 4:**
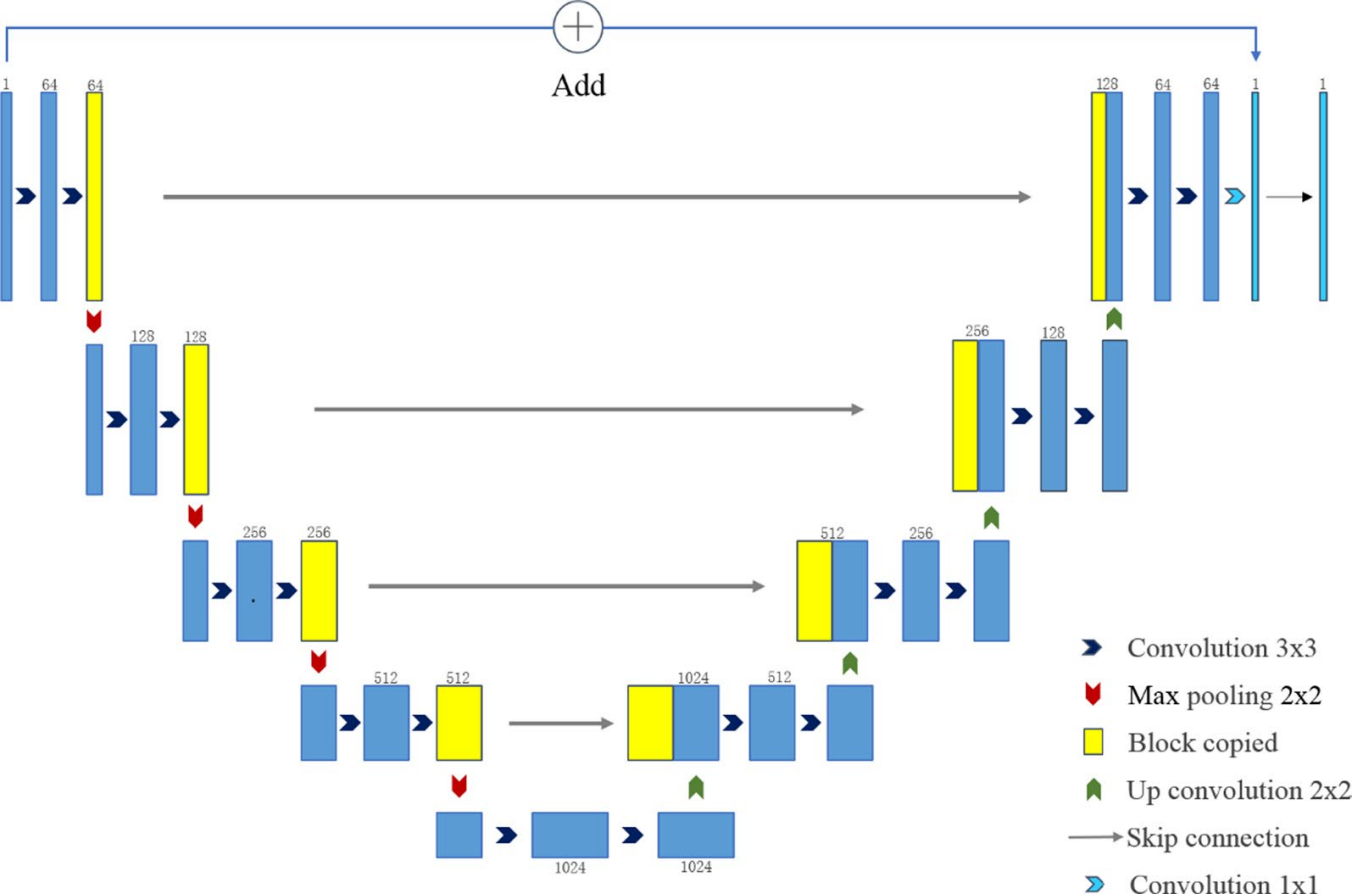
The schematic framework of the DL network

For training, we used U-net, which has been indicated to perform well in medical image processing [31]. U-net consists of encoder and decoder parts, as shown in Fig. 4. The encoder part contains five blocks, each of which contains two convolutional layers. The convolution kernel size is 3 × 3, and the activation function is the rectified linear unit (ReLU) function. Each block then implements the down-sampling through 2 × 2 max pooling. The decoder contains five blocks, each of which first performs a 2 × 2 up-sampling operation and a skip connection with the output of the block with the same resolution in the encoder part and then performs two 3 × 3 convolutions. The activation function used here is also the ReLU function. The input and the output of the last layer are superimposed as the output of the network.

The network's input was the dose distribution of the solid water (equivalent EPID) calculated by PRIMO. The size of the measured TI was 1024 × 768 and was resampled to 256 × 192 as the ground truth. The size of the dose distribution derived from PRIMO was 512 × 176, corresponding to 50 cm × 35.2 cm. To be consistent with the size of the EPID, a 40 cm × 30 cm area around the isocentre was intercepted and resampled to 256 × 192. The unit of the measured TI was the greyscale value. The loss function used in the training model was the mean square error (MSE). The Adam optimizer was used with a learning rate of 0.0001; default values were used for the other parameters. The training batch size was 12, and the model was trained 2000 times. The computer configuration was an i7-9700K CPU (Intel) and a GTX 2080 GPU (NVIDIA).

#### Analysis and verification method

There were 30 treatment plans with 150 IMRT beams used for patient treatments from various anatomic sites, including 10 lung cancers, 8 rectum cancers, 7 esophagus cancers, and 5 thymoma cancers. To prevent errors caused by changes in patient position, tumor size, patient weight, or organ motion during the actual treatment, these plans were transferred to different thicknesses (3, 5, 8, 10, 12, 15, 18, 20, 24 cm) of 40 cm × 40 cm (length × width) solid water phantoms and inhomogeneous CIRS thorax phantom (CIRS, Norfolk, VA). In total, 1500 field data were collected, of which 1200 fields were used as the training set, 150 fields were used as the validation set, and 150 fields were used as the test set. After training, the test data were input into the training model to predict the TI. The acquired EPID TI is not pre-processed for backscatter, so backscatter is included in both the training set and test set; the learning process of the DL network includes the EPID backscatter response. The predicted and measured TI was compared using global gamma analysis of 3%/3 mm and 2%/2 mm (5% threshold) to verify the model's accuracy.

To ensure that planning accuracy and dose delivery in the training data were correct, all plans were independently verified by Varian pre-treatment dose verification software (Portal dosimetry) and passed the global 3%/2 mm gamma evaluation by more than 98%.

#### Model sensitivity

To investigate the sensitivity of our prediction model to detect dose delivery errors in the phantom, several types of errors were deliberately introduced into the treatment plan and the phantom setup. The predicted TI for the unchanged plan and setup was compared to the measured TI for the delivery containing the errors using the global gamma analysis of 3%/3 mm (5% threshold). This was done using six IMRT treatment fields (2 lung plans, 2 rectum plans, 1 esophagus plan, and 1 thymoma plan) and followed the approach similar to the method of Bedford et al. [32] and Najem et al. [33]. The error plans (a–d) were delivered to the CIRS thorax phantom and error plan (e) was done using the 10 cm solid water phantom. The errors introduced as follow:

- Dose errors: The number of monitor units in all fields was increased by + 1% (Ea1), + 3% (Ea2), + 5% (Ea3), + 10% (Ea4) and − 5% (Ea5).
- Patient setup errors: The isocentre was shifted laterally towards the patient's right by 5 mm (Eb1), 10 mm (Eb2), and 20 mm (Eb3) and offset by 5 mm (Eb4), 10 mm (Eb5), and 20 mm (Eb6) in the anterior direction.
- Gantry angle error: The gantry angles were offset of + 5° (Ec1) and + 10° (Ec2).
- MLC errors: MLC leaves on all control points were opened by 5 mm (Ed1). Both banks shifted by 2 mm in the same direction (Ed2). The leaves of bank B that were within the field were shifted by 5 mm (Ed3). The central four MLC leaf-pairs on all control points were opened by 10 mm (Ed4).
- Change in phantom size: The original plan was delivered to the 10 cm solid water phantom, with 5 mm (Ee1), 10 mm (Ee2), 20 mm (Ee3) and 30 mm (Ee4) solid water added on top.

## Results

### Prediction model

**Fig. 5:**
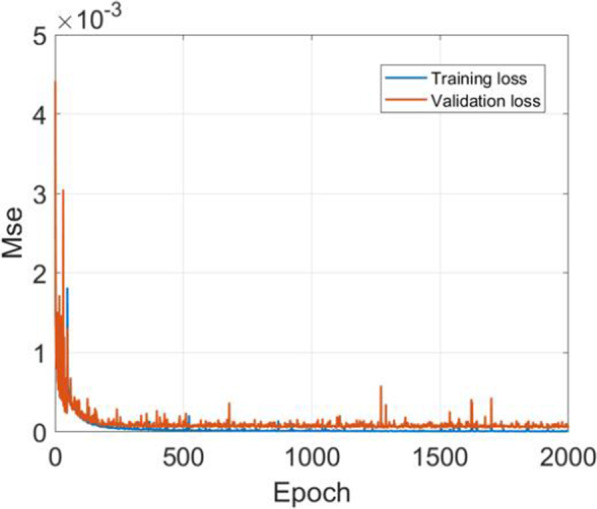
The loss curves of the training set (blue) and the validation set (orange)

It took approximately 40 h to train the network on a single GPU, and a total of 2000 training iterations were carried out. After each training, the validation dataset was used for verification to establish the performance of the network, and the loss values of the final training set and validation set were 8.3 × 10^–6^ and 4.4 × 10^–5^, respectively, as shown in Fig. 5.

**Fig. 6:**
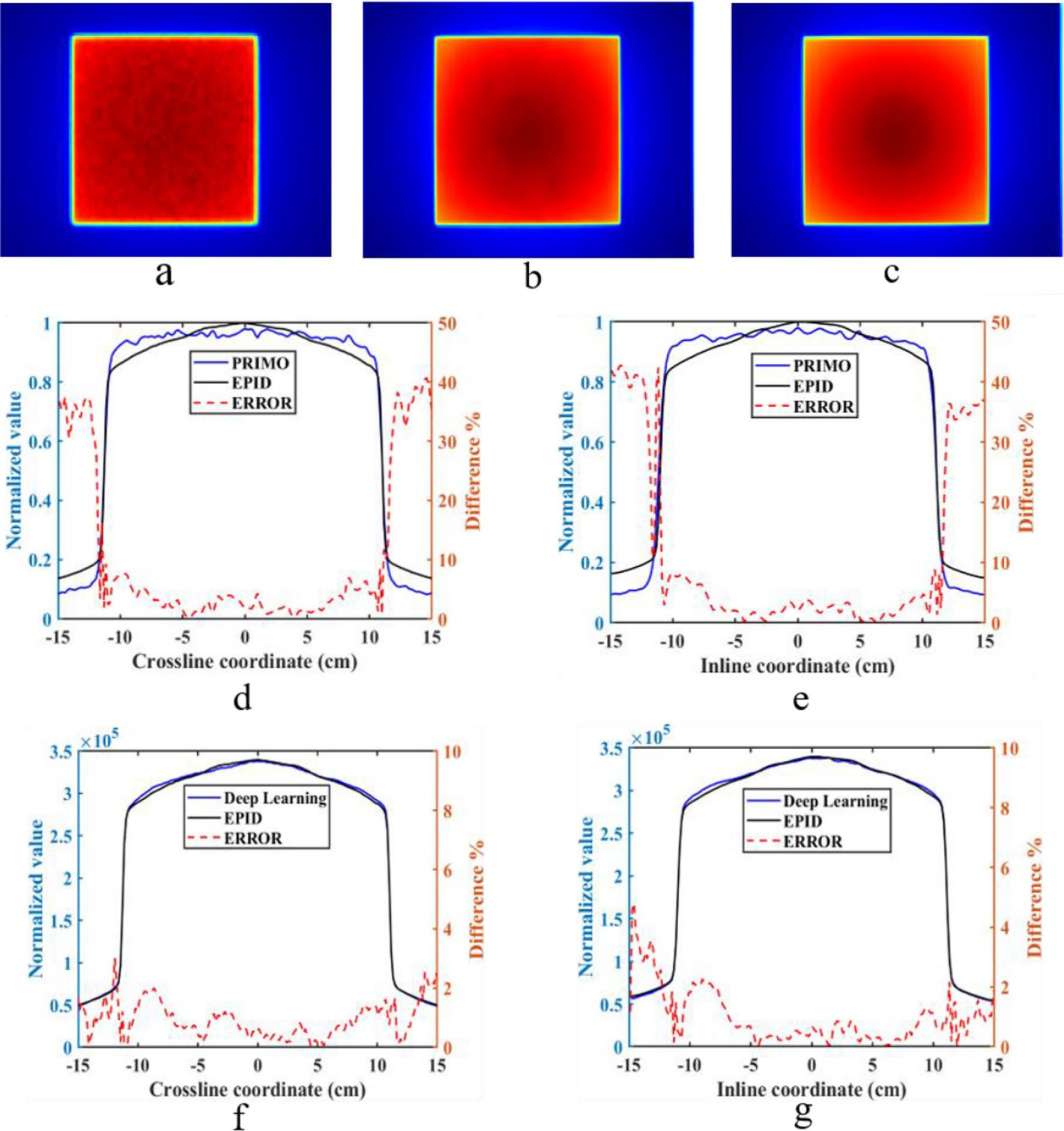
**a** The dose distribution of the equivalent EPID generated by PRIMO, **b** is the TI predicted by the DL network, **c** is the measured TI; **d** and **e** show the normalized dose distribution of the equivalent EPID, the normalized measured EPID TI and the relative error in crossline and inline directions; **f** and **g** show the predicted TI, the measured TI and the relative error in crossline and inline direction

We compared the image generated by PRIMO, the image predicted by the DL method and the actual measured image in the test set to verify the model's accuracy. Figure 6 shows the image when the solid water was 20 cm thick, the field size was 15 cm × 15 cm, and the gantry angle was 0°. The first row in Fig. 6 shows the image generated by PRIMO (Fig. 6a), the image predicted by the DL network (Fig. 6b), and the measured TI (Fig. 6c). In the image generated by PRIMO, the dose distribution of the equivalent EPID is affected by noise. The TI generated by the DL network converts the equivalent EPID dose map into the actual response of EPID and removes the influence of noise. The predicted image is consistent with the measured image.

Figure 6d, e show the comparison between the image calculated by PRIMO and the measured image in the crossline and inline directions, both of which have been normalized to their maximum values. As the off-axis distance increases, the error between them also increases, and the maximum error in the field is 10%. Figure 6f, g show the absolute value of the TI predicted by the DL network and the measured TI in the crossline and inline directions; the relative error in the field is less than 2%.

**Fig. 7:**
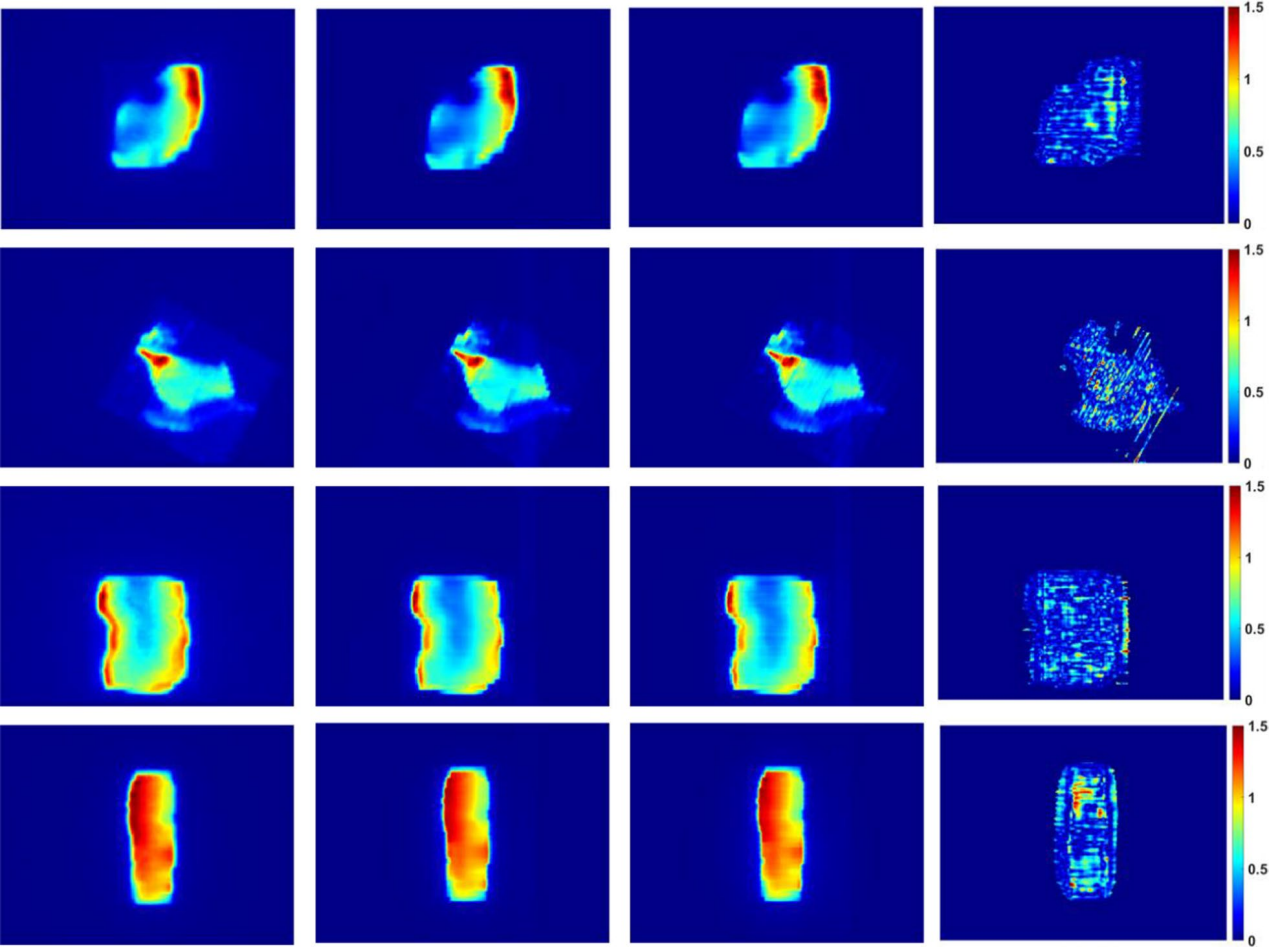
The dose distribution of the equivalent EPID (the first column), the prediction image (the second column), the EPID measurement image (the third column), and the 2%/2 mm gamma distribution map (the fourth column) corresponding to the four different fields (the first row is lung cancer field, the second row is rectum cancer field, the third row is thymoma cancer field and the fourth row is esophagus cancer field)

**Fig. 8:**
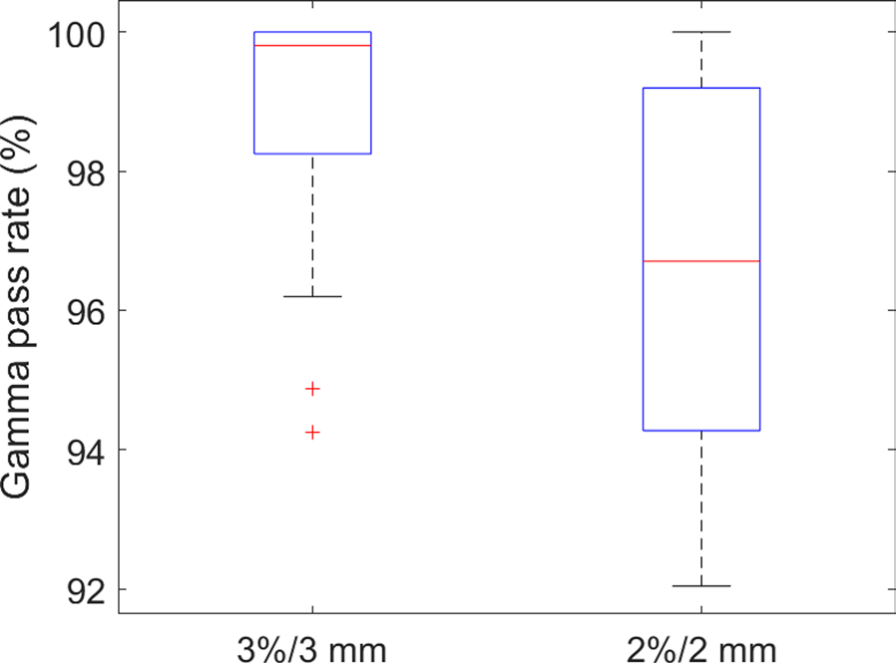
Gamma analysis of the test set

Standard global gamma analyses of 3%/3 mm and 2%/2 mm (5% threshold) were performed on the TI predicted by the DL network and the measured EPID TI in the test set. The gamma pass rates were greater than 96.7% and 92.3%, and the mean gamma values were 0.21 and 0.32, respectively. The predicted and measured values were in good agreement. Figure 7 shows the dose distribution of the equivalent EPID generated by PRIMO, the predicted TI, the measured TI, and the corresponding 2%/2 mm gamma distribution map of the four IMRT fields (1 lung cancer field, 1 rectum cancer field, 1 thymoma cancer field and 1 esophagus cancer field). Figure 8 shows the test set data corresponding to gamma pass rates of 3%/3 mm and 2%/2 mm.

### Model sensitivity

To verify the sensitivity of our prediction model to detection errors, we introduced errors to the treatment plan and phantom setup for comparison. The gamma indices for the unperturbed predicted TI with the perturbed measured TI are given in Table 1.

**Table 1:** The 3%/3 mm gamma pass rate for the error plans

Plan		Lung 1	Lung 2	Rectum 1	Rectum 2	Esophageal	Thymoma
No error		99.98	100.00	99.65	99.34	99.87	99.77
Dose error	Ea1	98.72	99.54	99.12	99.31	98.53	99.47
	Ea2	85.68	89.36	95.31	96.02	97.29	96.34
	Ea3	73.29	82.35	85.46	82.76	79.34	80.43
	Ea4	60.75	78.91	55.21	71.97	64.89	69.54
	Ea5	80.23	84.42	90.56	79.36	71.75	85.55
Setup error	Eb1	94.47	95.98	98.25	95.78	98.65	94.48
	Eb2	86.22	88.87	94.59	89.36	91.15	80.05
	Eb3	72.62	73.46	87.42	73.88	65.01	66.17
	Eb4	99.78	99.78	99.57	99.02	99.74	99.42
	Eb5	99.58	99.73	99.49	99.27	99.55	99.69
	Eb6	99.33	99.63	99.59	99.06	99.71	99.54
Gantry error	Ec1	97.91	99.50	91.04	98.31	98.52	97.83
	Ec2	93.22	95.26	78.04	97.41	89.26	88.43
MLC error	Ed1	4.07	4.92	5.97	5.20	6.35	6.19
	Ed2	92.13	80.48	92.55	95.57	88.28	94.84
	Ed3	14.23	26.41	22.13	21.97	16.81	13.12
	Ed4	77.44	83.80	87.20	70.80	74.04	75.78
Phantom size change	No error	100.00	99.75	99.21	99.42	100.00	98.97
	Ee1	99.95	98.26	97.34	99.13	99.54	98.64
	Ee2	93.18	94.21	91.64	93.64	94.15	94.31
	Ee3	76.16	80.15	74.97	82.35	71.07	79.29
	Ee4	55.22	50.17	64.32	48.23	40.43	53.97

From the results reported in Table 1, our prediction model should be sensitive to dose errors; when the dose error is greater than 3% (Ea2), the gamma pass rate begins to decrease. When the dose error is greater than 5% (Ea3), the gamma pass rate is less than 86%. For the setup error of the phantom, when the isocentre position shifted laterally towards the patient's right by 5 mm (Eb1), 10 mm (Eb2) and 20 mm (Eb3), the gamma pass rate decreased continuously, whereas the measured TI was unaffected by the anterior offset (Eb4–Eb6). For the gantry angle error, when the gantry angle is offset by 5° (Ec1), the result changes slightly, and when the gantry angle is offset by 10° (Ec2), the gamma pass rate decreases significantly. The results of MLC errors described in Table 1 demonstrate that our prediction model is sensitive to a range of MLC errors, various types of MLC errors have different effects on gamma pass rate, and the pass rate is significantly reduced. We used the same method as Najem et al. [33] to add additional solid water slabs to the 10 cm solid water phantom to verify the sensitivity of our model to patient outline changes. When the added slabs were larger than 10 mm (Ee2), the gamma pass rate is less than 95%. In summary, our prediction model is sensitive to dose errors, some types of setup errors, MLC errors, and phantom size changes. It is weakly sensitive to gantry angle errors.

## Discussion

In this study, we converted the equivalent EPID dose value calculated by PRIMO into the actual EPID response through a DL network and compared it with the measured EPID TI during treatment for the in vivo treatment verification of IMRT. The results show that this method has the potential to be used clinically for in vivo treatment verification.

When using the MC method to simulate the EPID response [6, 10], the process needs to model the structure of the EPID, which is usually industrial secrets and requires relatively specialized knowledge. The current version of the PRIMO software is used to calculate the dose of the phantom or patient, and the phantom or patient model could be imported from an external DICOM-CT file or create a slab phantom with the material composition internally. We do not have detailed structural and compositional material information of the EPID. In addition, the PRIMO contains limited materials, and some materials that comprise EPID are not included (e.g., the Scintillator layer composed of Gd202S: Tb) and do not support user-defined materials, so it is difficult to simulate EPID using the full PRIMO model directly. Additionally, the need for long computation time has been a major obstacle to using MC methods in clinical treatment verification. In our study, the complex EPID was replaced with homogeneous solid water and combined with the plan CT, and then the combined CT was imported into PRIMO to calculate the dose distribution. Our method does not require separate modelling of the EPID, and thus is simpler and more convenient than other methods. Furthermore, only EPID TI was acquired during treatment and there was no need to execute the treatment plan repeatedly, so the dose verification process saves the time of the linac. The calculation time in our model is mainly to calculate the dose value of equivalent EPID, which is related to the number of histories used in the MC simulation process and computer configuration. In our simulation, the phase-space file contains 6.88 × 10^9^ histories. For an IMRT field with 110 control points, the calculation process takes approximately 10 min, and it only takes approximately 5 s to predict EPID TI with the trained deep learning network.

The EPID off-axis response is different from the linac output, and the beam profile "horns" were removed after dark field and flood field correction. Moreover, the inverse square ratio reduced the signal, and the X-ray was attenuated after passing through the phantom and air, resulting in fewer particles reaching the equivalent EPID plane. The statistical uncertainty of the MC method increased, and the water dose calculated by MC was affected by noise. Therefore, the dose value calculated by PRIMO is different from the actual EPID response and cannot be converted into the EPID TI by simple linear scaling (Fig. 6). We modelled the conversion relationship between the dose value and the EPID response through the DL network. Due to the user-friendly and simple MC modelling process of PRIMO and the modelling potential power of the DL network, this model can quickly and accurately use the MC algorithm for in vivo treatment verification.

The reported results from Table 1 show that our model is weakly sensitive to the gantry angle error and isocentre position shifted anteriorly. These results agreed with the results reported by Bedford et al. [32] and Najem et al. [33]. The sensitivity of the model to those errors mainly depends on the anatomy of the patient. When the gantry angle is offset by 5° and the isocentre position is offset in the anterior direction, the equivalent path length of the ray passing through the phantom does not change dramatically and has little influence on the EPID TI. Suppose there are significant inhomogeneities in and around the treatment field; in that case, our prediction model has the potential to detect such errors that Bedford et al. [32] and Najem et al. [33] not detected (such as Eb1, Eb2, Eb3 and Ec2).

Deshpande et al. [34] also inserted solid water into plan CT to create a water-equivalent EPID and input it into the TPS to calculate the dose distribution. The dose distribution of the water-equivalent EPID calculated by the TPS was compared with the measured dose for verification. However, due to the large weight of the water-equivalent EPID, their model could not be rotated in the integrated gantry, and the treatment angle could only be set to 0° for verification. Consequently, the phantom could only be used to replace the patient for pre-treatment dose verification, and it is impossible to detect the error caused by the gantry rotation during treatment. In our model, the data collected during treatment include the actual gantry angle, thus eliminating this problem and allowing in vivo treatment verification.

Theoretically, our method could reverse convert the EPID TI to the dose in water. However, the inverse square ratio reduced the signal, and fewer particles reached the equivalent EPID plane. The equivalent EPID dose calculated by MC is affected by noise. If a noisy dose map is used as the label of the DL model, it will affect the results of dose verification. In the future, we will try to obtain the dose value as the label of the DL model through other methods (such as calculated by TPS or measured by the dose measurement tool) and convert the EPID TI to the water dose value.

PRIMO can import the patient dose distribution calculated by the TPS into the software and compare it with the dose distribution calculated by PRIMO [18]. However, this process verifies only the accuracy of the TPS calculation and fails to detect errors during the treatment process. In addition, PRIMO can use the DynaLog file to recalculate the dose distribution of the patient and compare it with the result calculated by the TPS for in vivo treatment verification [19]. This method cannot detect errors caused by patient positioning. In our study, EPID images were acquired during treatment; if the patient position changed, the predicted and measured images would be different. Therefore, our model has the potential to detect errors caused by plan transmission, patient positioning, and machine failure, which provides a new method for in vivo treatment verification using PRIMO.

## Conclusion

We developed a DL-based method to convert the dose value of solid water calculated by the MC method into the EPID response during the treatment process and compared it with the measured EPID TI for two-dimensional in vivo treatment verification. Because of the complexity of MC method modelling, achieving acceptable accuracy is time-consuming, which has prevented wider clinical application of the MC method. Our proposed method makes it simpler and faster to use the MC algorithm (PRIMO) to simulate the EPID response while ensuring calculation accuracy to facilitate in vivo radiotherapy treatment verification.

## Data Availability

The datasets during the current study are not publicly available due to some research that has not been completed, but is available from the corresponding author on reasonable request.

## References

[1] Das IJ, Cao M, Cheng CW, Misic V, Scheuring K, Schule E, Johnstone PA (2011). A quality assurance phantom for electronic portal imaging devices. J Appl Clin Med Phys.

[2] Greer PB (2005). Correction of pixel sensitivity variation and off-axis response for amorphous silicon EPID dosimetry. Med Phys.

[3] van Zijtveld M, Dirkx M, Breuers M, de Boer H, Heijmen B (2009). Portal dose image prediction for in vivo treatment verification completely based on EPID measurements. Med Phys.

[4] Olaciregui-Ruiz I, Rozendaal R, Mijnheer B, van Herk M, Mans A (2013). Automatic in vivo portal dosimetry of all treatments. Phys Med Biol.

[5] Gustafsson H, Vial P, Kuncic Z, Baldock C, Denham JW, Greer PB (2011). Direct dose to water dosimetry for pretreatment IMRT verification using a modified EPID. Med Phys.

[6] Fuangrod T, Woodruff HC, van Uytven E, McCurdy BM, Kuncic Z, O'Connor DJ, Greer PB (2013). A system for EPID-based real-time treatment delivery verification during dynamic IMRT treatment. Med Phys.

[7] Camilleri J, Mazurier J, Franck D, Dudouet P, Latorzeff I, Franceries X (2016). 2D EPID dose calibration for pretreatment quality control of conformal and IMRT fields: a simple and fast convolution approach. Phys Med.

[8] van Elmpt W, Nijsten S, Petit S, Mijnheer B, Lambin P, Dekker A (2009). 3D in vivo dosimetry using megavoltage cone-beam CT and EPID dosimetry. Int J Radiat Oncol Biol Phys.

[9] Wendling M, McDermott LN, Mans A, Olaciregui-Ruiz I, Pecharroman-Gallego R, Sonke JJ, Stroom J, van Herk M, Mijnheer BJ (2012). In aqua vivo EPID dosimetry. Med Phys.

[10] Chytyk-Praznik K, VanUytven E, vanBeek TA, Greer PB, McCurdy BM (2013). Model-based prediction of portal dose images during patient treatment. Med Phys.

[11] Van Uytven E, Van Beek T, McCowan PM, Chytyk-Praznik K, Greer PB, McCurdy BM (2015). Validation of a method for in vivo 3D dose reconstruction for IMRT and VMAT treatments using on-treatment EPID images and a model-based forward-calculation algorithm. Med Phys.

[12] Mijnheer B, Beddar S, Izewska J, Reft C (2013). In vivo dosimetry in external beam radiotherapy. Med Phys.

[13] Berry SL, Sheu RD, Polvorosa CS, Wuu CS (2012). Implementation of EPID transit dosimetry based on a through-air dosimetry algorithm. Med Phys.

[14] Siebers JV, Kim JO, Ko L, Keall PJ, Mohan R (2004). Monte Carlo computation of dosimetric amorphous silicon electronic portal images. Med Phys.

[15] Chytyk K, McCurdy BM (2009). Comprehensive fluence model for absolute portal dose image prediction. Med Phys.

[16] Rodriguez M, Sempau J, Brualla L (2013). PRIMO: a graphical environment for the Monte Carlo simulation of Varian and Elekta linacs. Strahlenther Onkol.

[17] Fogliata A, Stravato A, Reggiori G, Tomatis S, Wurfel J, Scorsetti M, Cozzi L (2018). Collimator scatter factor: Monte Carlo and in-air measurements approaches. Radiat Oncol.

[18] Paganini L, Reggiori G, Stravato A, Palumbo V, Mancosu P, Lobefalo F, Gaudino A, Fogliata A, Scorsetti M, Tomatis S (2019). MLC parameters from static fields to VMAT plans: an evaluation in a RT-dedicated MC environment (PRIMO). Radiat Oncol.

[19] Rodriguez M, Brualla L (2019). Treatment verification using Varian's dynalog files in the Monte Carlo system PRIMO. Radiat Oncol.

[20] Bacala AM (2020). Linac photon beam fine-tuning in PRIMO using the gamma-index analysis toolkit. Radiat Oncol.

[21] Zhou J, Peng Z, Song Y, Chang Y, Pei X, Sheng L, Xu XG (2020). A method of using deep learning to predict three-dimensional dose distributions for intensity-modulated radiotherapy of rectal cancer. J Appl Clin Med Phys.

[22] Kontaxis C, Bol GH, Lagendijk JJW, Raaymakers BW (2020). DeepDose: towards a fast dose calculation engine for radiation therapy using deep learning. Phys Med Biol.

[23] Xing Y, Nguyen D, Lu W, Yang M, Jiang S (2020). Technical Note: A feasibility study on deep learning-based radiotherapy dose calculation. Med Phys.

[24] Fan J, Xing L, Dong P, Wang J, Hu W, Yang Y (2020). Data-driven dose calculation algorithm based on deep U-Net. Phys Med Biol.

[25] Babier A, Mahmood R, McNiven AL, Diamant A, Chan TCY (2020). Knowledge-based automated planning with three-dimensional generative adversarial networks. Med Phys.

[26] Zhang S, Wang H, Tian S, Zhang X, Li J, Lei R, Gao M, Liu C, Yang L, Bi X (2021). A slice classification model-facilitated 3D encoder-decoder network for segmenting organs at risk in head and neck cancer. J Radiat Res.

[27] Valdes G, Solberg TD, Heskel M, Ungar L, Simone CB (2016). Using machine learning to predict radiation pneumonitis in patients with stage I non-small cell lung cancer treated with stereotactic body radiation therapy. Phys Med Biol.

[28] Zhen X, Chen J, Zhong Z, Hrycushko B, Zhou L, Jiang S, Albuquerque K, Gu X (2017). Deep convolutional neural network with transfer learning for rectum toxicity prediction in cervical cancer radiotherapy: a feasibility study. Phys Med Biol.

[29] Li J, Wang L, Zhang X, Liu L, Li J, Chan MF, Sui J, Yang R (2019). Machine learning for patient-specific quality assurance of VMAT: prediction and classification accuracy. Int J Radiat Oncol Biol Phys.

[30] Wang L, Li J, Zhang S, Zhang X, Zhang Q, Chan MF, Yang R, Sui J (2020). Multi-task autoencoder based classification-regression model for patient-specific VMAT QA. Phys Med Biol.

[31] Ronneberger O, Fischer P, Brox T. U-Net: convolutional networks for biomedical image segmentation. In: International Conference on Medical image computing and computer-assisted intervention: 2015. Springer; 2015. p. 234–41.

[32] Bedford JL, Hanson IM, Hansen VN (2014). Portal dosimetry for VMAT using integrated images obtained during treatment. Med Phys.

[33] Najem MA, Tedder M, King D, Bernstein D, Trouncer R, Meehan C, Bidmead AM (2018). In vivo EPID dosimetry for IMRT and VMAT based on through-air predicted portal dose algorithm. Phys Med.

[34] Deshpande S, Blake SJ, Xing A, Metcalfe PE, Holloway LC, Vial P (2018). A simple model for transit dosimetry based on a water equivalent EPID. Med Phys.

